# Immobilization of an Endo-β-*N*-acetylglucosaminidase for the Release of Bioactive *N-*glycans

**DOI:** 10.3390/catal8070278

**Published:** 2018-07-10

**Authors:** Joshua L. Cohen, Sercan Karav, Daniela Barile, Juliana M. L. N. de Moura Bell

**Affiliations:** 1 Department of Food Science and Technology, University of California, One Shields Avenue, Davis, CA 95616, USA; jlcohen@ucdavis.edu (J.L.C.); dbarile@ucdavis.edu (D.B.); 2 Department of Molecular Biology and Genetics, Canakkale Onsekiz Mart University, 17100 Canakkale, Turkey; sercankarav@comu.edu.tr; 3 Foods for Health Institute, University of California, One Shields Avenue, Davis, CA 95616, USA; 4 Department of Biological and Agricultural Engineering, University of California, One Shields Avenue, Davis, CA 95616, USA

**Keywords:** *N*-glycans, mass spectrometry, immobilization, prebiotic, glycosidase, recombinant, kinetic, nano-LC Chip Q-ToF MS

## Abstract

As more is learned about glycoproteins’ roles in human health and disease, the biological functionalities of *N*-linked glycans are becoming more relevant. Protein deglycosylation allows for the selective release of *N*-glycans and facilitates glycoproteomic investigation into their roles as prebiotics or anti-pathogenic factors. To increase throughput and enzyme reusability, this work evaluated several immobilization methods for an endo-β-*N*-acetylglucosaminidase recently discovered from the commensal *Bifidobacterium infantis*. Ribonuclease B was used as a model glycoprotein to compare *N*-glycans released by the free and immobilized enzyme. Amino-based covalent method showed the highest enzyme immobilization. Relative abundance of *N*-glycans and enzyme activity were determined using matrix-assisted laser desorption/ionization time-of-flight mass spectrometry. Kinetic evaluation demonstrated that upon immobilization, both V_max_ and the K_m_ decreased. Optimal pH values of 5 and 7 were identified for the free and immobilized enzyme, respectively. Although a higher temperature (65 vs. 45 °C) favored rapid glycan release, the immobilized enzyme retained over 50% of its original activity after seven use cycles at 45 °C. In view of future applications in the dairy industry, we investigated the ability of this enzyme to deglycosylate whey proteins. The immobilized enzyme released a higher abundance of neutral glycans from whey proteins, while the free enzyme released more sialylated glycans, determined by nano-LC Chip Q-ToF MS.

## 1. Introduction

Glycoproteins are a biologically important class of components with modulatory roles in signaling and cell adhesion. *N*-linked glycans are covalently bound to an asparagine residue with the consensus sequence on the primary structure of asparagine-X-serine/threonine (with X representing any amino acid besides proline) [1]. The attachment is mediated by the reducing end *N*-acetylglucosamine moiety of the glycan onto the asparagine residue [2]. All *N*-glycans share a common trimannosyl, chitobiose (two β-1,4 linked *N*-acetylglucosamine residues) core and are classified based on how the core is elongated with various monosaccharides. *N*-glycans can be decorated with *N*-acetylneuraminic acid or *N*-glycolylneuraminic acid (sialylated) and/or fucose (fucosylated) in various antennary combinations to give rise to a heterogeneity of combinations, even on a single glycosylation site. Glycans can be classified based on their antennary decorations, with neutral, fucosylated, sialylated, and both fucosylated and sialylated being the primary non-plant glycans.

As a model for understanding the interplay of indigestible carbohydrates and beneficial gut bacteria, human milk oligosaccharides (HMOs) have been widely studied and found to exhibit uniquely effective prebiotic functionalities [3]. While HMOs are quite abundant in human milk (up to 15 g/L in colostrum), their commercial-scale isolation from human milk is not feasible [4]. The activities and roles of intact and released *N*-linked glycans upon human consumption are not well understood due, in part, to the lack of adequate deglycosylation and analytic methods to release, identify, and quantity the released glycans [5]. *N*-glycans can be released from the protein moiety using harsh chemical treatments or enzymatic methods, the latter allowing for the recovery of intact protein and glycans. Deglycosylating enzymes include peptide-*N*-glycosidases and endo-β-*N*-acetylglucosaminidases, which differ in cleavage points of the core of *N*-glycans [6].

Due to striking similarities in structures and functions, released *N*-linked glycans from bovine milk glycoproteins have been studied for their ability to promote the growth of commensal bacteria in vitro [7,8]. A recently discovered endo-β-*N*-acetylglucosaminidase (Endo-BI-1), isolated from *B. infantis*, was shown to deglycosylate human milk glycoproteins more effectively than other endo-β-*N*-acetylglucosaminidase isolated from several commensal *Bifidobacterium* species [9]. More importantly, released *N*-glycans by this novel enzyme from bovine milk proteins displayed a remarkable selective prebiotic activity on *Bifidobacterium longum* subsp. *infantis* in vitro, and may promote the growth of other beneficial bacteria while inhibiting pathogens [7,8,10]. However, to establish *N*-glycans as an alternative source of prebiotic carbohydrates to human milk, further research into the selectivity of bovine *N*-glycans and their functional similarity with HMOs with respect to commensal, beneficial, and pathogenic gut organisms is necessary.

Whey, the co-product of cheese manufacture, is a potential commercially available source of underutilized glycoproteins. The concentration of proteins in bovine cheese whey ranges from 1 to 2%, wherein approximately 4–9% of those proteins are *N*-glycosylated immunoglobulins, lactoferrin, and transferrin [11,12]. Lactoferrin, while found in substantial quantities in human milk and bovine colostrum, is present in bovine milk and cheese whey at the trace level.

Yet, the nearly 200 million tonnes of whey produced each year globally [13] conservatively translates to 2 million tonnes of whey proteins, corresponding to approximately 100,000 tonnes of bovine milk glycoproteins available for subsequent processing. With such a large availability of glycoproteins, the development of large-scale processing strategies to release and isolate *N*-glycans for further characterization and elucidation of their biological functionalities becomes a key step for further commercialization of these compounds.

Indeed, enzyme immobilization may facilitate reaction scale-up considering the possibility for broad reactor systems and catalyst reusability. Enzyme immobilization is the process wherein a soluble enzyme is attached or adsorbed onto a solid support, entrapped within a matrix, or otherwise aggregated enabling enzyme reuse during production [14]. However, widespread industrial use of immobilized enzyme remains limited due to a perceived loss of enzyme activity, lack of universal immobilization techniques, and cost implications [15,16]. In general, enzyme immobilization facilitates the separation of products and catalyst where removal from the final product to terminate the reaction and to reuse the catalyst is important in producing pure bioactive molecules. Covalent, adsorption, entrapment, and aggregation methods of immobilization have their inherent advantages and disadvantages in a reactor system. These factors are based on substrate size and ability to access the enzyme active site, physicochemical stability, and applicability in industrial processes [15]. Although the use of immobilized enzymes has been validated by the food industry to a limited extent [17], the use of glycosidases has been restricted to lab-scale [6].

The overall goal of this study was to evaluate several types of immobilization methods and resins for a novel endo-β-*N*-acetylglucosaminidase (Endo-BI-1) isolated from *B. infantis.* In addition to the development of an immobilization method based on protein immobilization yield and enzyme activity, the effects of the immobilization method on pH and temperature sensitivity of the immobilized enzyme were evaluated. Enzyme reusability was also evaluated. Significant efforts were directed towards the development of sensitive and high-throughput analytical methods: in particular, MALDI-ToF MS was used to study the rate and specificity of the deglytosylation reaction kinetics on RNase B, and nano-LC-Chip Q-ToF MS was used to characterize the diverse pool of *N*-glycans released from bovine colostrum whey proteins using free end immobilized enzyme.

## 2. Results and Discussion

### 2. 1. Protein Immobilization Yield

**Figure 1.**
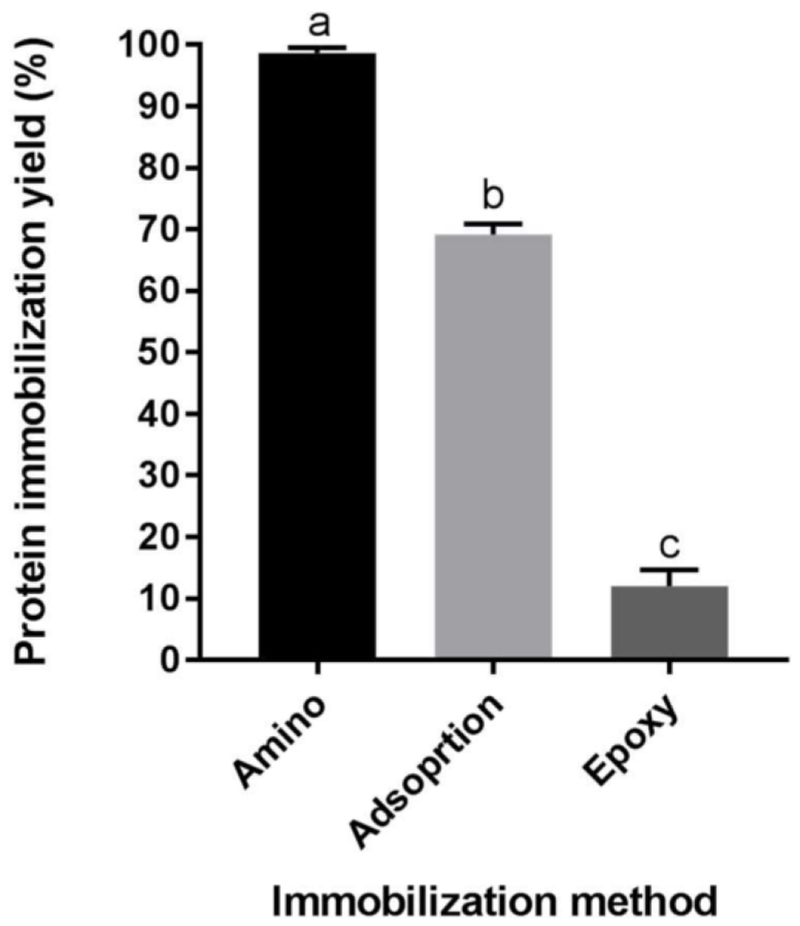
Protein immobilization yield for amino, adsorption, and epoxy immobilization methods, measured fluorometrically by Qubit Protein Assay Kit. Error bars represent one standard deviation and means followed by different letters (a, b, and c) are statistically different at *p* < 0.05.

Enzyme immobilization yield of amino-, epoxy- and adsorption-immobilized Endo BI-1 was evaluated by quantifying the unbound protein remaining in the supernatant and resin washing buffer post-immobilization. The fluorescence method employed herein (Qubit Protein Assay Kit) demonstrated the high immobilization yield (>98%) of the amino-based resin, whereas divinylbenzene (DVB)-based hydrophobic interaction adsorption immobilized 70% of the enzyme (Figure 1). Epoxy-based covalent binding yielded the lowest immobilization yield at 12%. These results were corroborated by SDS-PAGE, where denser Endo BI-1 bands in the supernatants were present in epoxy and adsorption methods, while amino-based immobilization had no visible band (Figure S1). Differences between immobilization yields for covalent and adsorption immobilization methods have been observed previously and can possibly be attributed to the strength of interaction between hydrophobic interactions and covalent bonds [18]. However, low binding using epoxide-activated resin may be due to non-optimal methods to identify the ideal pH, time, and temperature for increased binding [19]. Further investigation into those conditions may improve protein immobilization yield for the epoxide-activated resin.

### 2.2. Relative Quantification of N-glycans by MALDI-ToF MS

An additional goal of this research was to identify a sensitive, specific, and rapid method to determine relative quantities of *N*-glycans to facilitate assessment of enzyme activity. Spectrophotometric methods involving phenol-sulfuric acid have been used, but lack selectivity for certain saccharide types and tend to be affected by other compounds present in the sample, not only the *N*-glycan products of this reaction [20–22]. Additionally, the diverse array of monosaccharides present in *N*-glycans adds a layer of complexity leading to inconsistent responses in these assays [21]. As an alternative, matrix-assisted laser desorption/ionization time-of-flight mass spectrometry MALDI-ToF MS has been used for relative quantification of various carbohydrates [23,24]. It is known that MALDI-ToF, albeit rapid and effective in providing results, may suffer from low reproducibility due to inconsistent crystallization of the samples on the target plate, leading to shot-to-shot variation. These disadvantages can be overcome by using an internal standard [25]. Additionally, MALDI has been used to study enzymatic reactions including both large and small substrates/products [26–28].

**Figure 2.**
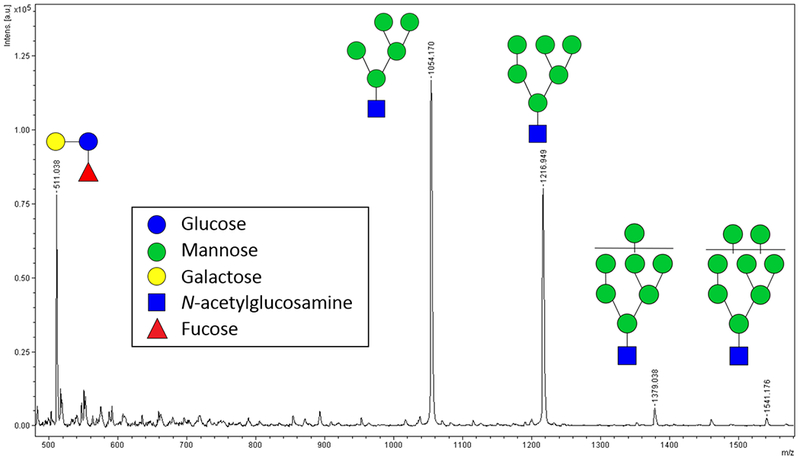
MALDI-ToF mass spectrum for 3-FL (*m/z* 511)-spiked *N*-glycans released from RNase B using Endo BI-1.

Linearity of the 3-FL (3′-fucosyllactose)-spiked released *N*-glycan system was evaluated. An example of a typical annotated mass spectrum of *N*-glycans can be seen in Figure 2. The theoretical *m/z* for sodiated 3-FL, Man 5, Man 6, Man 7, and Man 8 are 511.16, 1054.34, 1216.40, 1378.45, and 1540.50 respectively, accounting for one fewer *N*-acetylglucosamine residue according to cleavage by Endo BI-1. The most abundant glycan released from RNase B was Man5 (*m/z* 1054), with larger glycans being less abundant, which was consistent with previous literature [29]. Bovine RNase B was chosen as a model glycoprotein due to its single *N*-glycosylation site and straightforward glycan composition.

Over the examined order of magnitude range, the normalized response plotted against *N*-glycan/I.S. (internal standard) ratio was linear (R^2^ = 0.995, Figure S2). A high R^2^ value indicates that analysis of released *N*-glycans in presence of I.S. analyzed by MALDI-ToF MS is a suitable tool for rapid relative quantification of *N*-glycans needed for evaluating kinetic parameters over a substantial range of concentrations and amounts of reactant.

### 2.3. Comparing Immobilized Enzyme Activities

**Figure 3.**
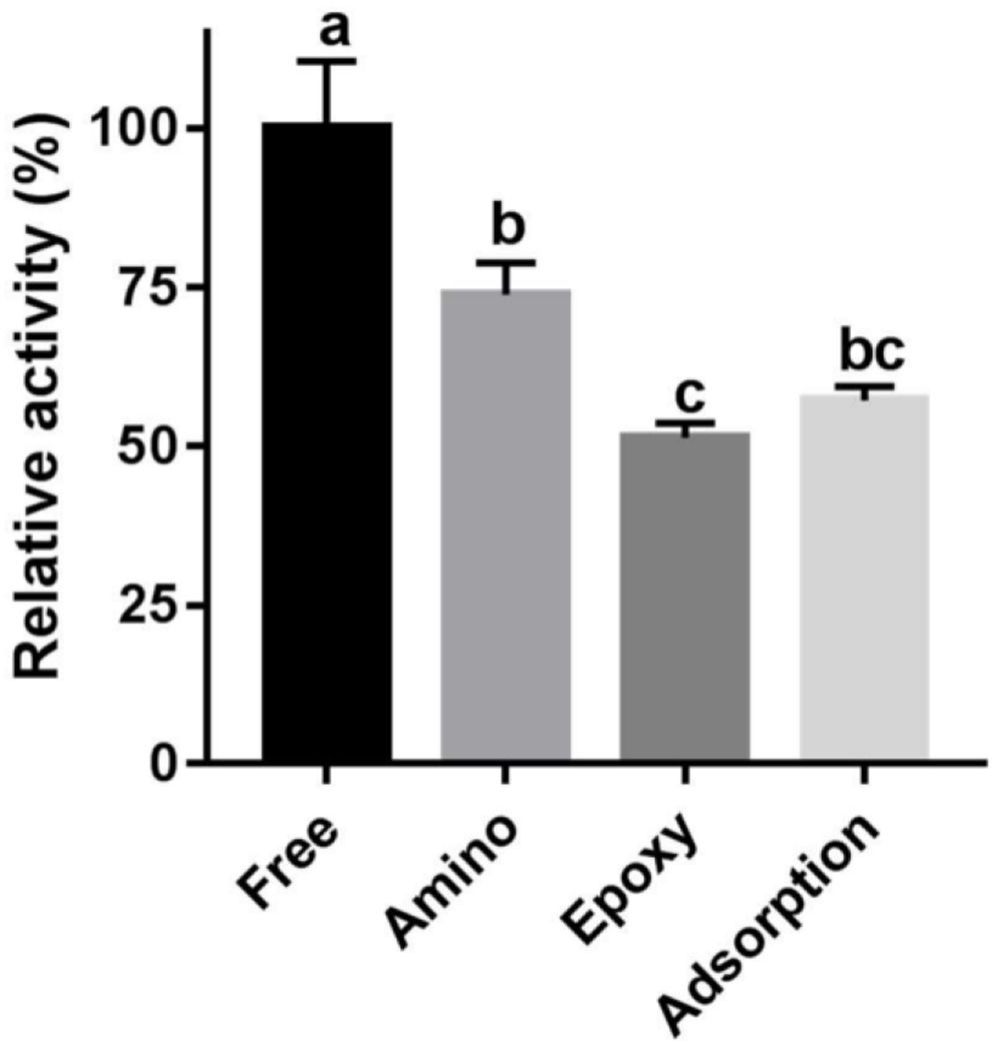
Relative activity of free or immobilized Endo BI-1 (on amino, epoxy, and adsorption resins) on the glycoprotein RNase B. Error bars represent one standard deviation. Means followed by different letters (a, b, and c) are statistically different at *p* < 0.05.

The activity of the enzyme immobilized by each method (amino, epoxy, and adsorption) was evaluated on RNase B, and reacted for 90 min at 45 °C and pH 5.0. The normalized relative abundance of released *N*-glycans was determined by MALDI-ToF MS, with activity based on the amount of glycans released by the free enzyme (Figure 3). Free enzyme had the highest activity, with amino, epoxy, and adsorption methods retaining 73, 51, and 57% activity, respectively. Diminished activity upon immobilization is common, and has been reported previously with glutaraldehyde-mediated binding and hydrophobic adsorption [30]. A combination of diffusional/steric hindrances and possible binding and occlusion of enzyme active sites may explain the reduction in enzyme activity [16]. Additionally, heterogeneous catalysis impacts enzyme activity. The amino-based immobilization was selected for all subsequent experiments due to its higher activity compared with epoxy and adsorption methods, as well as its improved catalyst density on the resin.

### 2.4. Temperature and pH Sensitivity of Immobilized Enzyme Using RNase B

**Figure 4.**
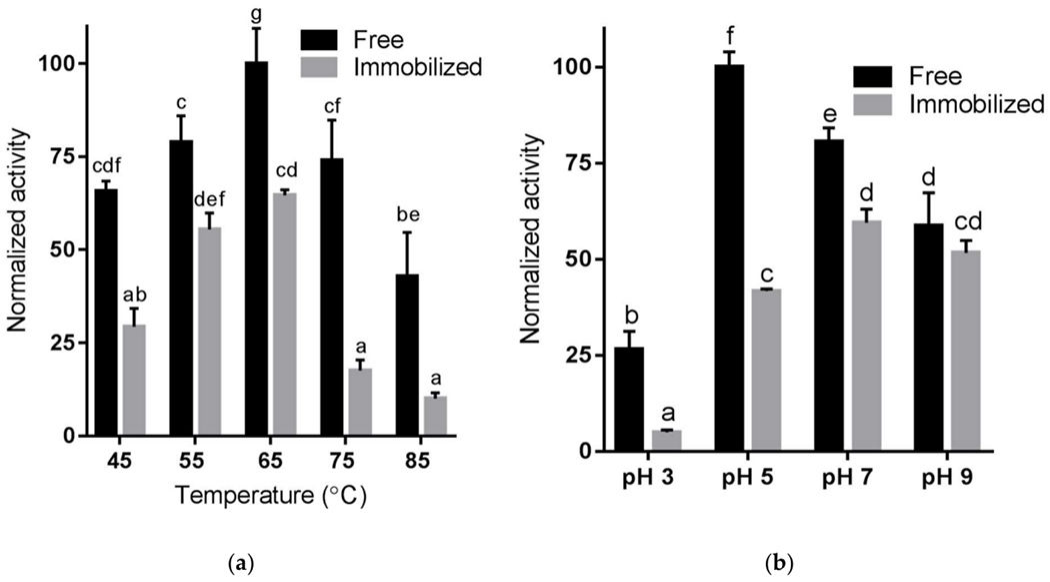
Mean relative activity of free and immobilized enzyme at different (**a**) temperature and (**b**) pH values using RNase B. Error bars represent one standard deviation and means, within the same chart, followed by different letters (a, b, c, d, e, and f) are statistically different at *p* < 0.05.

Enzyme properties can change with respect to pH and temperature sensitivity when immobilized [31,32]. Temperature sensitivity was evaluated at pH 5 with 20 mM Na_2_HPO_4_ buffer at temperatures from 45 to 85 °C (Figure 4a). The optimal temperature for both free and immobilized Endo BI-1 was 65 °C. These results differ from what was previously reported by Karav et al. [33], in which a lower optimum temperature of 52 °C was identified for the free enzyme. This discrepancy could be attributed to different reaction conditions used in both studies. While RNase B and a short reaction time (20 min) were used in the present work, bovine colostrum whey protein and longer reaction times (15–475 min) were evaluated in the previous study. At the optimum temperature identified (65 °C), the immobilized enzyme retained 63% of the activity compared with the free enzyme. In each experimental condition of both temperature and pH, the activity of immobilized Endo BI-1 was lower than that of the free enzyme, at all conditions. For both free and immobilized enzymes, activities decreased at temperatures above 65 °C.

To evaluate pH sensitivity, 20 mM Na_2_HPO_4_ buffer was adjusted to pH 3, 5, 7, and 9. Reactions with free and immobilized Endo BI-1 and RNase B were carried out at 45 °C for 20 min, and the normalized activity can be seen in Figure 4b. The optimal pH for free Endo BI-1 was 5, in agreement with previous reports [9], while for the immobilized enzyme a shift to a neutral pH (7.0) was observed. At the optimal pH for the free (pH 5) and immobilized enzyme (pH 7), the immobilized enzyme retained approximately 62% of the activity of the free enzyme at 20 min of reaction. However, the immobilized enzyme was more resilient in the range of pH 7 to 9, where reduced differences in the activities of the free and immobilized enzyme were observed. Indeed, at pH 9, the activities of free and immobilized Endo BI-1 were not statistically different (Figure 4a). Our results are in agreement with several reports in the literature where a shift on the working pH of the immobilized enzyme to a neutral or more alkaline pH was observed, with the same being attributed to changes in the amine group during the covalent binding [31,34]. In the context of scaling up *N*-glycan release using bovine milk proteins, the optimum pH of the immobilized enzyme (pH 7), presents a clear advantage over the free enzyme, considering that milk and dairy streams naturally have a pH close to neutral and would not necessitate the use of buffers for pH adjustment, which can become problematic and costly at large scale. Literature suggests that immobilization alters temperature and pH sensitivity by stabilizing the enzyme conformation and creating stable microenvironments around the enzyme or close to its active site [35]. The data here presented provides valuable information for future scale-up of the process and testing other substrates for glycan release.

### 2.5. Enzyme Kinetics Using RNase B

**Figure 5.**
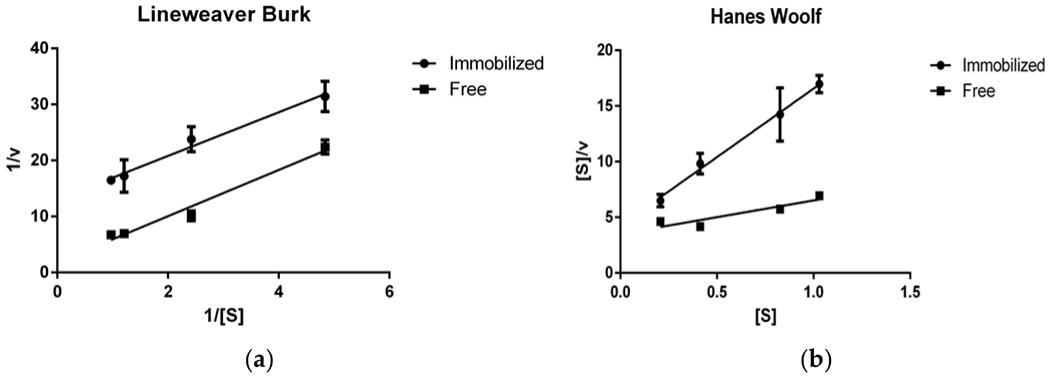
Lineweaver Burk (1/[*S*] (mL/mg) vs. 1/*v* (mL × min/mg)) (**a**) and Hanes–Woolf ([*S*] (mg/mL) vs. *s*/*v* (min)) (**b**) linearized plotting techniques for the estimation of kinetic parameters of free and immobilized EndoBI-1 on RNase B.

**Figure 6.**
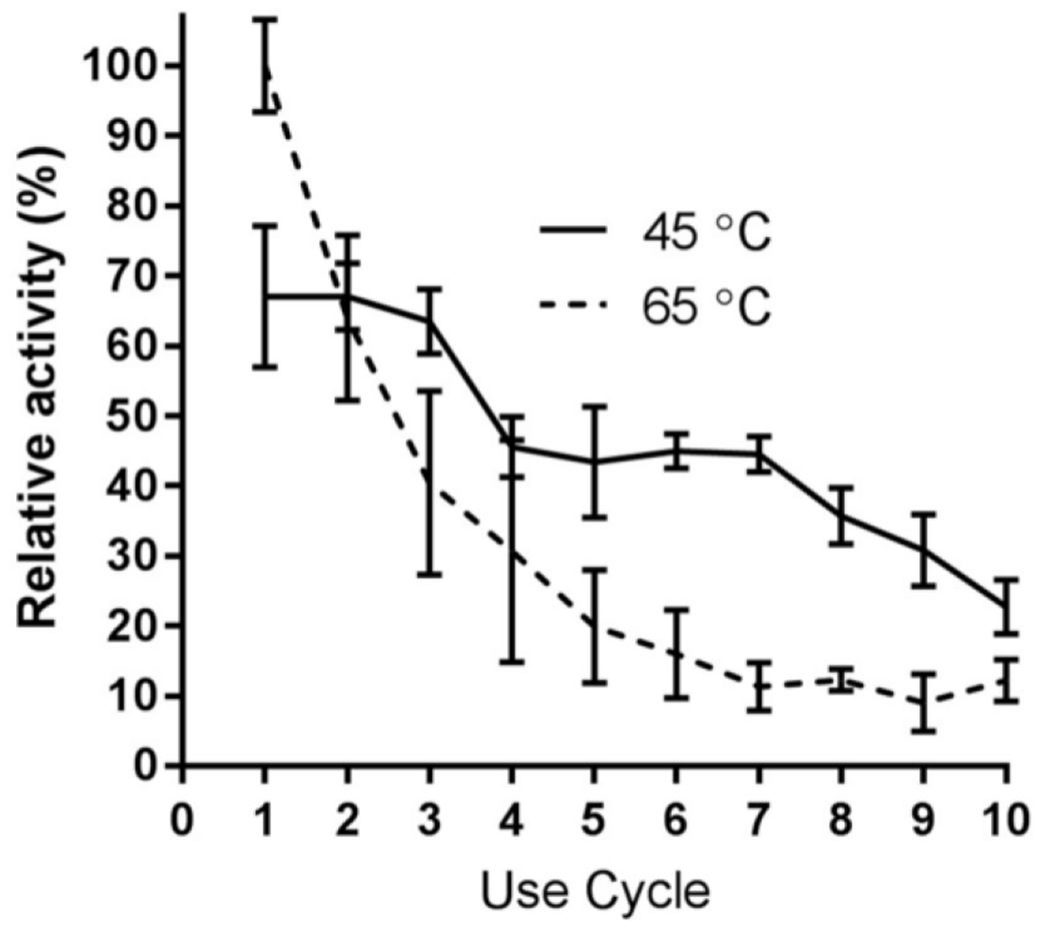
Mean relative activity of immobilized Endo BI-1 on RNase B following several reuse cycles. Error bars represent one standard deviation.

To gain more quantitative insight into the mechanistic changes of the enzymatic deglycosylation upon immobilization, the kinetic parameters (V_max_, K_m_) were estimated using linearized plotting techniques (Lineweaver-Burk and Hanes Woolf) to reduce the error of non-linear estimation (Figure 5). V_max_, the maximum forward velocity of the reaction, decreased from 0.551 glycan/min to 0.076 glycan/min (Lineweaver Burk estimation) upon immobilization of Endo BI-1 as calculated on RNase B (Figure 6). The Michaelis constant (K_m_, a measure of substrate affinity) also decreased upon immobilization from 2.27 mg/mL for the free enzyme to 0.299 mg/mL once Endo BI-1 was immobilized on the amino-activated methacrylate resin. The goodness-of-fit for the Lineweaver Burk linear model (R^2^) for free and immobilized enzyme was 0.89 and 0.96 respectively. Hanes Woolf linear estimation revealed similar trends (0.333 and 0.081 V_max_ values, 1.17 and 0.345 K_m_ values for free and immobilized enzymes, respectively) to Lineweaver Burk. Decreased V_max_ is typical of enzyme immobilization [32,36], wherein lower maximum velocity is likely due to limited access of the large, macromolecular substrate to the active site of the immobilized enzyme. The immobilized enzyme is on a molecular tether, and steric hindrances limit access to more occluded glycan sites on the substrate with respect to the three-dimensional structure. As a point of comparison, β-1,4-galactosidase, with a molecular weight of approximately 465 kDa in its tetrameric form, with lactose as substrate at 360 Da has an overall substrate/enzyme size ratio of 0.0008:1, whereas for RNase B and Endo BI-1, the substrate/enzyme size ratio is approximately 0.32:1. This suggests a more limited diffusion and increased steric challenge with respect to active site availability.

Increased apparent affinity accompanied by decreased V_max_ is consistent with uncompetitive inhibition. Possible explanations of this phenomenon are the formation of a more stable enzyme-substrate complex between Endo BI-1 and RNase B upon immobilization, or simply deglycosylated RNase B may have difficulty diffusing away from immobilized Endo BI-1 upon interaction with the active site. Both stable enzyme-substrate complexes and diffusional challenges would increase apparent affinity and inhibit forward reaction progress and are documented phenomena [36,37]

Enzymes with diverse, macromolecular substrates may exhibit substrate-dependent kinetic behavior. For example, when Endo BI-1 was tested on different glycoproteins, V_max_ and K_m_ also varied. The Michaelis constants for RNase B, lactoferrin, and concentrated whey proteins were 0.25, 0.43, and 0.90 mg/mL respectively, potentially due to different sizes, purities, and physicochemical properties of the glycoproteins [20]. The overall increase in K_m_ for Endo BI-1 in this study (0.33 vs. 025 mg/mL) is likely related to differences in reaction conditions and analytical methods used in both studies. While this study utilized MALDI-ToF MS to measure the hydrolytic efficiency of Endo BI-1, previous studies utilized a spectrophotometric phenol-sulfuric assay, wherein the diverse array of monosaccharides present in *N*-glycans from lactoferrin and other whey proteins could have yielded different responses.

### 2.6. Immobilized Enzyme Reusability with RNase B

A substantial advantage of enzyme immobilization is the potential reusability of the catalyst. Amino-mediated immobilized Endo BI-1 was subjected to ten 20-min reuse cycles at 45 and 65 °C to determine the effects of different temperatures on the activity and reusability of the immobilized enzyme (Figure 6). Enzyme activity was higher at 65 °C compared with 45 °C. At 45 °C, the immobilized enzyme activity corresponded to approximately 65% of the enzyme activity at 65 °C. Despite higher enzyme activity at 65 °C, a higher decrease in the enzyme activity was observed after each reuse cycle. At 65 °C, enzyme activity was reduced to 63% of its original value after one cycle of reuse, and by the sixth use cycle, it had been reduced to and stabilized at 10 to 15% of its original activity. Conversely, at 45 °C, the enzyme activity remained consistent up to three reuse cycles. Despite the initial higher enzyme activity at 65 °C, from the third cycle onward reactions at 45 °C had greater residual enzyme activity than reactions performed at 65 °C. These results indicate that although 65 °C is optimal for initial glycan release, it does not favor repeated enzyme use for longer reaction times. It is possible to reuse the immobilized enzyme up to seven cycles at 45 °C with significant retention of its activity (50% from the initial activity). Our results are in agreement with several reports in the literature, where the use of lower temperatures (e.g., 37 °C) has favored high reusability, even though higher temperatures may favor more rapid catalysis [38,39]. Regarding an industrially-relevant enzyme with macromolecular substrates, Chauhan reported a loss of over 85% of polygalacturonase activity after 6 cycles using adsorption onto celite [40]. Additionally, reaction time and temperature affect the selective release of *N*-glycans from more complex and diverse glycoproteins, which may determine the choice of the reaction temperature thus affecting reusability of the immobilized enzyme [41]. Importantly, fouling with a protein-based substrate may also impact reusability [42].

### 2.7. Release of *N*-glyans from Bovine whey Proteins

**Figure 7.**
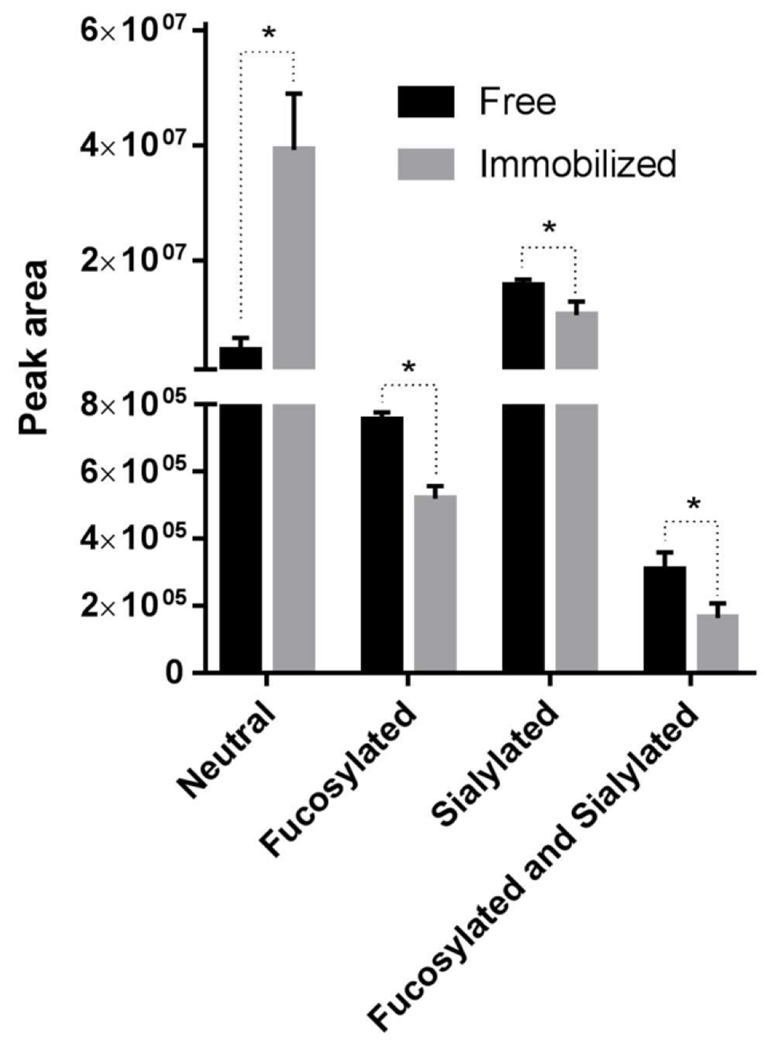
Mean relative quantities of various classes of released *N*-glycans from bovine colostrum whey proteins determined by nano-LC Chip Q-ToF MS/MS by free and immobilized Endo BI-1. Error bars represent one standard deviation. * Represents significant differences within the same class of *N*-glycans at *p* < 0.05.

Cheese whey, a co-product of the dairy industry, is a source of *N*-linked glycoproteins including lactoferrin, lactoperoxidase, transferrin, and various immunoglobulins [12]. Free and immobilized Endo BI-1 was incubated with whey proteins purified from bovine colostrum at pH 7 at 45 °C for 1 h. The glycans were purified and characterized by nano LC-Chip Q-ToF mass spectrometry and each compound’s relative abundance was determined and further classified based on the class of glycan to which each compound belongs (Figure 7).

Overall, twenty-six unique *N*-glycan structures were identified and confirmed by tandem mass spectra. The *N*-glycans were characterized based on their monosaccharide composition, which included neutral (containing only hexose and *N*-acetylhexosamine residues), sialylated, fucosylated, and fucosylated and sialylated. For neutral glycans the immobilized enzyme released eight times higher relative amounts than free Endo BI-1. For the class fucosylated and sialylated, the free enzyme released significantly higher amount of glycans (approximately double) compared with the free enzyme. Free enzyme released 50% more of each fucosylated and sialylated glycan class. For glycans with both sialylation and fucosylation, the free enzyme released nearly double the relative amount of glycans compared with immobilization. It is worth mentioning that fucosylated and sialylated glycans were present in lowest amounts. Both sialylated and neutral glycans represented the most abundant glycan types released from colostrum whey proteins. Importantly, unique glycan compositions should only be compared between samples, and not among the various *N*-glycan structures themselves. In fact, differences in ionization efficiency due to diverse monosaccharide composition hinder direct comparisons in the absence of identical pure standards.

The *N*-glycan structures released by Endo BI-1 were tabulated according to the glycan composition notation Hex_HexNAc_Fuc_NeuAc_NeuGc, which correspond with the number of hexose, *N*-acetylhexosamine, fucose, *N*-acetylneuraminic acid, and *N*-glycolylneuraminic acid residues respectively (Table SI). The most abundant glycans released from colostrum whey proteins by free Endo BI-1 were 4_3_0_1_0 and 3_3_0_1_0 while the most abundant glycan structures released by immobilized Endo BI-1 were 3_3_0_0_0 and 5_1_0_0_0 (Man5). The relative abundance for released 3_3_0_0_0 and 5_1_0_0_0 for immobilized enzyme compared with free enzyme were 27 and 14 times higher, respectively. These large differences are the main drivers of the substantial differences in relative abundance of neutral glycans. Both 3_3_0_0_0 and 4_3_0_1_0 were identified as the most abundant glycans released from a similar protein source using Endo BI-1 [41]. Although high mannose compounds like 5_1_0_0_0 and 6_1_0_0_0 were previously found in the bovine and human milk *N*-glycome, likely deriving from bovine lactoferrin, they were not previously described as being released from bovine colostrum whey proteins with free Endo BI-1 [41,43].

Despite the many technological breakthroughs, deglycosylation is still not a well-characterized process. Recently, the kinetics of the commercial enzyme peptide-*N*-glycosidase F (PNGase F) were investigated to understand the impact of sialylation on the release of *N*-glycans [44]. Presence of sialic acid slowed glycan release, while fucosylation also affected the deglycosylation rate. Because RNase B contains a single glycosylation site and high mannose type glycans, these differences would not be observed. However, when using a different enzyme (Endo BI-1) and complex substrates like bovine colostrum whey proteins under different conditions, kinetics and deglycosylation behavior would likely be substantially different. Additionally, the presence of methacrylate resin with pore diameters within the range of 300 to 600 Å may facilitate binding of small, neutral glycans such as 3_3_0_0_0 and 5_1_0_0_0 or the protein regions containing them, favoring their release compared with free Endo BI-1. Antennary moieties such as fucose or sialic acid could also change physicochemical interactions between the glycan and the methacrylate supports as well as the glycan with the immobilized enzyme itself. The ability to modulate glycan release based on reaction conditions has been previously described by Le Parc et al. [41]. Additional changes on the glycan release resulting from the immobilization method used suggests a high degree of complexity concerning enzymatic deglycosylation.

## 3. Materials and Methods

### 3.1. Enzyme Production

Recombinant endo-β-(1,4)-*N*-acetylglucosaminidase (Endo BI-1) from *Bifidobacterium longum* subsp. *infantis* ATCC 15697 from the University of California Davis Viticulture and Enology Culture Collection (Davis, CA, USA) was cloned into *E. coli* using a pEcoTM-T7-cHis cloning kit (GeneTarget Inc, San Diego, CA, USA). Endo BI-1 was expressed and isolated in a stock containing 65 mg/mL enzymebased upon previous literature [33].

### 3.2. Enzyme Immobilization

Endo BI-1 was immobilized onto methacrylate resin according to manufacturer’s directions (Purolite, Llantrisant, Wales, UK). Epoxide-activated (Lifetech ECR8204F), amino-activated (Lifetech ECR8309F), and divinylbenzene (DVB, Lifetech ECR1030M) adsorption resins were used in this study. Covalent (epoxide- and amino-activated resins) and adsorption (divinylbenzene resin) immobilization techniques were selected in this study due to limited macromolecular substrate diffusion through entrapment matrices [45].

To activate the amino resin, 2% glutaraldehyde in 1× PBS as a crosslinking agent was added to the resin prior to enzyme addition and incubated for 1 h, after which it was washed with 0.1× phosphate-buffered saline (PBS) to remove excess glutaraldehyde. Endo BI-1 enzyme stock was added to each activated resin (50 mg enzyme per gram activated resin, wet basis) in buffer mass equal to four times the mass of the resin. For epoxide-activated immobilization, resin was incubated with enzyme in 10× PBS buffer for 42 h. For adsorption-based immobilization, DVB resin was incubated with enzyme for 18 h in 0.1× PBS buffer. Amino-based immobilization was incubated with enzyme in 0.1× PBS for 24 h, based on the manufacturers’ recommendations. Enzyme incubation took place at 25 °C and 100 rpm and each immobilization method was performed in triplicate. Following incubation, resins were washed with four volumes of 0.1× PBS buffer. The wash buffer was collected and further analyzed to determine the protein immobilization yield.

### 3.3. Evaluating Protein Immobilization Yield

Wash buffers from enzyme immobilization were treated with four volumes of −20 °C ethanol and held at −30 °C for 60 min to precipitate proteins, after which the samples were centrifuged at 4000× *g* for 30 min at 4 °C. The supernatant was discarded, and the pellet was resuspended in nanopure water. The total protein was loaded to the immobilization resin and the protein remaining in the wash buffer was measured. The protein immobilization yield was determined by the following equation:

$$\text{Protein immobilization yield}\;(\%)=\frac{P_{L}-P_{w}}{P_{L}}\times 100\%$$

where P_L_ is the mass of protein loaded and P_w_ is the mass of protein in the resin washing buffer after immobilization [46]. Total protein was measured fluorometrically in triplicate using the Qubit Protein Assay Kit (Life Technologies, Grand Island, NY, USA). Purified protein samples were also analyzed by sodium dodecyl sulfate polyacrylamide gel electrophoresis (SDS-PAGE) on 4–15% precast polyacrylamide gels to visualize amount of protein not bound to immobilization resin [9].

### 3.4. N-glycan Analysis by MALDI-ToF Mass Spectrometry

A microflex MALDI-ToF mass spectrometer (Bruker Daltonics GmbH, Bremen, Germany) was used. Ten μL of purified *N*-glycan samples were spiked with five μL 100 mg/L of the human milk oligosaccharide 3′-fucosyllactose (3-FL, Dextra Laboratories Inc., Reading, UK) as an internal standard. A one μL aliquot of spiked sample was combined with an equal volume of 2,5-dihydroxybenzoic acid matrix (20 mg/mL in 30% acetonitrile, 0.1% trifluoroacetic acid) and 0.4 μL 1 mM NaCl was added to achieve ionization in positive mode. 0.5 μL spiked sample with matrix and NaCl was spotted in duplicate onto a ground steel target plate and dried under vacuum. Mass calibration was conducted using a polysaccharide ladder extracted from beer [47].

Ionization was carried out using a 337.1 nm laser, and detected in reflectron positive mode with a reflector voltage of 20.02 kV in the mass range of 300 to 2500 *m/z.* The final spectra were the sum of 2000 laser shots. MS intensities of four glycans (namely Man 5, Man 6, Man 7, Man 8) released from bovine ribonuclease B (RNase B, Sigma Aldrich, St. Louis, MO, USA) were normalized to the MS intensity of 3-FL internal standard, summed, and considered the normalized relative abundance of glycan.

Linearity of the MALDI-ToF method was determined by preparing a stock of glycans released from RNase B using free Endo BI-1. Following purification as described above, varying amounts of released *N*-glycan was added to a constant amount of 3-FL internal standard and analyzed by MALDI-ToF MS as described above. The summed, normalized intensity was plotted against the volume of *N*-glycans added and a linear regression was fit to the data.

### 3.5. Preliminary Comparison of Immobilization Methods Efficiency Using Ribonuclease (RNase) B

Accounting for varying immobilization yields of the enzyme onto the beads, resin containing 40 μg immobilized Endo BI-1 or 40 μg free enzyme was reacted with 600 μg in 20 mM Na_2_HPO_4_ buffer for 90 min at 45 °C at pH 5. The reaction was terminated with ethanol at −30 °C and glycans were purified and characterized as described in the MALDI-ToF MS analysis section. Summed, normalized MS intensities for the four most abundant glycans were normalized to 100% activity for the free enzyme. One-way analysis of variance (ANOVA) was used along with Tukey’s multiple comparisons test with GraphPad Prism 7 (Graphpad, La Jolla, CA, USA) to determine the statistical significance of the data at *p* < 0.05.

### 3.6. Effect of Immobilization on Enzyme Resilience to pH and Thermal Changes

To identify the optimum temperature for the immobilized enzyme, RNase B was incubated in pH 5 buffer as described previously and free or immobilized enzyme was added [9]. The reaction mixture (enzyme + substrate) was incubated for 20 min at temperatures ranging from 45 to 85 °C. Their respective deglycosylating activities were normalized and compared.

The pH of 20 mM Na_2_HPO_4_ buffer was adjusted to 3, 5, 7, and 9 using 1 N HCl or NaOH. Free and immobilized Endo BI-1 was reacted with RNase B at 45 ° C at each pH for 20 min and the relative activity, measured as the enzyme ability to deglycosylate RNase B, was measured by MALDI-ToF MS. Experiments were conducted in triplicate and a two-way ANOVA was used along with Tukey’s multiple comparisons in GraphPad Prism 7 to determine statistical significance of the data at *p* < 0.05.

### 3.7. Determination of Kinetic Parameters of Enzyme Using RNase B

Relevant kinetic parameters relating to maximum reaction velocity (V_max_) and the Michaelis-Menten constant (k_m_) were determined by measuring the normalized glycan intensity at varying substrate concentrations (0.1 to 1.1 g/L) in 20 mM Na_2_HPO_4_ buffer under optimal pH and temperature conditions for the free and immobilized enzyme. Aliquots were taken in four-minute intervals up to 20 min. Linearized plotting (Lineweaver Burk and Hanes Woolf) were utilized to calculate K_m_ and V_max_ [48,49].

### 3.8. Evaluating Reusability of Immobilized Endo BI-1 Using RNase B

Reusability of immobilized Endo BI-1 was evaluated for ten cycles of 20 min at both 65 °C and 45 °C in presence of RNase B. Between each cycle, the supernatant from the reaction was removed and subsequently treated with cold ethanol for protein precipitation and glycan isolation. After each reaction cycle, the remaining resin containing the immobilized enzyme was washed with a 20 mM Na_2_HPO_4_ buffer at a 4:1 ratio (buffer/reaction volume) and fresh substrate-containing buffer was added for the next reaction cycle. Enzyme activity after the first reaction was considered as 100%. Experiments were conducted in triplicate.

### 3.9. Release of *N*-glycans from Bovine Colostrum whey Proteins by Free and Immobilized Endo BI-1

Bovine colostrum whey proteins were isolated from bovine colostrum whey kindly provided by La Belle (Bellingham, WA, USA). Bovine colostrum whey proteins were isolated and purified using a 10 kDa molecular weight cut-off ultrafiltration as described previosly by Karav et al. [33] and were deglycosylated by free and immobilized Endo BI-1 in triplicate. Bovine colostrum whey protein concentration was determined by Qubit fluorometric kit according to manufacturer’s instructions as described previously. Briefly, Endo BI-1 (free or immobilized) was added to purified colostrum whey proteins at a ratio of 1:75 in 20 mM Na_2_HPO_4_ buffer at pH 7 and reacted for 1 h at 45 °C based on previous reports [33]. The reaction was terminated, and proteins were precipitated using cold ethanol. The supernatant was dried by vacuum centrifuge and subsequently resuspended, whereupon glycans were purified from contaminants using C18 cartridges (Glygen, Columbia, MD, USA) and desalted by porous graphitized carbon (PGC, Glygen) solid phase extraction as described previously [7,50]. Eluate from PGC was dried, resuspended in nanopure water for subsequent nano-LC-Chip Q ToF analysis.

### 3.10. N-glycan Analysis by Mass Spectrometry Nano-LC-Chip Q ToF

Samples were transferred to polypropylene vials and 6 μL were injected into an Agilent 6520 nano-LC-Chip quadrupole time-of-flight mass spectrometer (Q-ToF MS, Agilent Technologies, Santa Clara, CA, USA). Glycans were further purified on a 9 mm × 75 μm PGC enrichment column and then separated on a 43 mm × 75 μm PGC analytical column with binary solvents, gradient, and data collection based on Karav et al. [7]. The following minor modifications were applied: scan range for MS was 450 to 2500 *m/z,* and for MS/MS was 100 to 3200 *m/z.* Spectra in both MS and MS/MS modes were collected at a rate of 0.63 spectra/s. The collision energy in the collision cell corresponded to a slope of 1.8/100 Da.

Chromatograms from the nano-LC-Chip Q ToF were curated and compounds identified in Masshunter Qualitative Analysis software (version B.07.00 Agilent Technologies) using a previously described bovine milk *N*-glycan bioinformatic library adjusted for one fewer *N-*acetylglucosamine in the chitobiose core. The library assumed compositions including hexose (Hex), *N*-acetylhexosamine (HexNAc), fucose (Fuc), *N*-acetylneuraminic acid (NeuAc), and *N*-glycolylneuraminic acid (NeuGc) [43]. Additionally, structures included a tetrasaccharide core containing three hexose (e.g., mannose) moieties and one HexNAc (*N*-acetylglucosamine) moiety. Structures were confirmed using MS/MS fragmentation patterns and isotopic distribution. A library of glycan structures specific to the sample set was assembled based on retention time, molecular formula and confirmed *m/z.* Relative abundances of *N*-glycans were obtained using Masshunter Profinder software (version B.06.00, Agilent Technologies). The batch targeted feature extraction algorithm with the following parameters was applied using the generated database: minimum abundance of 750 counts, charge state up to +3, retention time window of 2 min, and a glycan isotope model. Peak areas were verified for accurate integration and isotopic distribution.

## 4. Conclusions

These findings highlight the advantages and limitations of different enzyme immobilization strategies, in particular when considering an enzyme targeting the release of glycans from macromolecular substrates such as glycoproteins. The immobilization process shifted the optimum pH of the enzyme to a neutral pH, which is of relevance when considering the immobilization of this enzyme for industrial applications, such as in dairy products or other food products having a pH close to neutral. The use of moderate temperatures favored the reuse of the immobilized enzyme. Immobilized enzyme reusability may compensate for its initial reduced activity. When using a complex and diverse source of glycoproteins as bovine colostrum, the immobilized enzyme released a higher abundance of neutral *N*-glycans than the free enzyme, which yielded a different overall composition of glycans by class. This difference suggests that specific glycan release can be achieved by potentially tailoring immobilized enzyme reactions conditions in future studies.

Further investigation of the different rates of glycan release, possibly due to limited enzyme accessibility to specific glycan sites on glycoproteins, may enhance our understanding of the role of certain glycosylation sites and structures on their function, in particular for therapeutic glycoproteins such as monoclonal antibodies. Investigation on alternative glycoprotein substrates of plant origin is warranted to understand substrate specificity. This study presents a scalable technique for enzymatic deglycosylation of industrially relevant glycoproteins. Optimized reactor configurations, including flow-through column reactors and fluidized beds, could favor glycan release and subsequent isolation from deglycosylated proteins in downstream isolation, enabling future processing scale-up and commercialization of bioactive glycans and novel therapeutics.

## Supplementary Material

Supplemental
