# Supplementary material for: Immobilization of an Endo-β-N-acetylglucosaminidase for the Release of Bioactive N-glycans
Source: Catalysts. Author manuscript; Available in PMC 2019 Mar 15. (PMC6419974; doi:10.3390/catal8070278)
Supplement: Supplemental [file NIHMS990122-supplement-Supplemental.docx]

Article

**Immobilization of an Endo-β-*N*-Acetylglucosaminidase for the Release of Bioactive *N*-Glycans**

Joshua L. Cohen ^1^, Sercan Karav ^2^, Daniela Barile ^1,3^ and Juliana M.L.N. de Moura Bell ^1,4^*

**^1^** Department of Food Science and Technology, University of California, One Shields Avenue, Davis, CA 95616, USA; jlcohen@ucdavis.edu (J.L.C); dbarile@ucdavis.edu (D.B.)

**^2^** Department of Molecular Biology and Genetics, Canakkale Onsekiz Mart University, 17100 Canakkale, Turkey; sercankarav@comu.edu.tr

**^3^** Foods for Health Institute, University of California, One Shields Avenue, Davis, CA 95616, USA

**^4^** Department of Biological and Agricultural Engineering, University of California, One Shields Avenue, Davis, CA 95616, USA

***** Correspondence: jdemourabell@ucdavis.edu; Tel.: +1-530-752-5007

Received: 01 June 2018; Accepted: 04 July 2018; Published: date


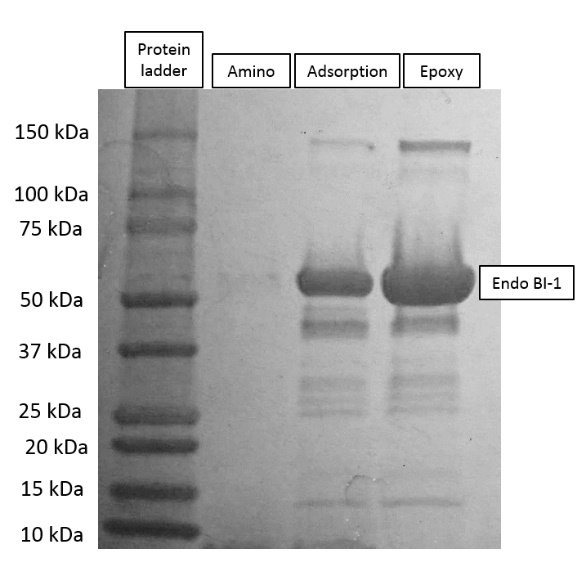


**Figure S1.** Sodium dodecyl sulfate polyacrylamide gel electrophoresis of immobilization washings from amino, adsorption, and epoxy methods on a 4–15% acrylamide gel. Endo BI-1 weighs approximately 53 kDa.


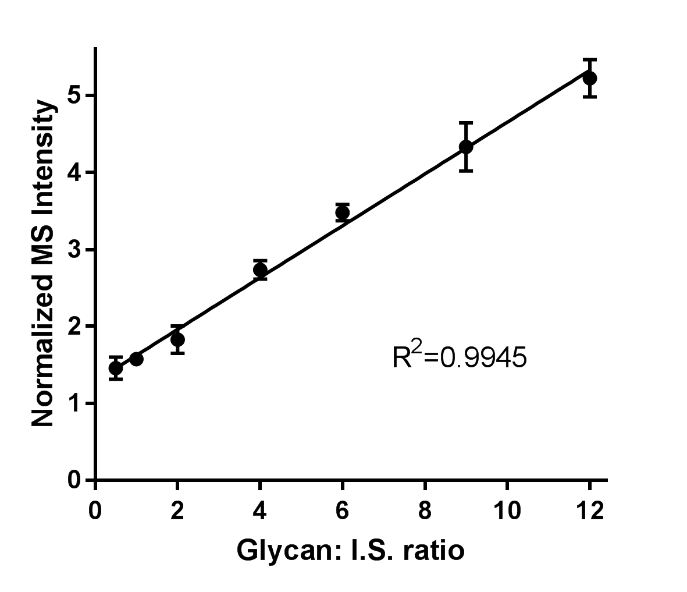


**Figure S2.** Normalized MALDI-ToF MS intensity vs. N glycan: I.S. ratio for glycans released from RNase B. A linear regression was applied, with error bars representing one standard deviation.

**Table S1.** Mean relative quantities (±S.D.) of *N*-glycans released by free and immobilized Endo BI-1 from bovine colostrum whey proteins as determined by nano-LC Chip Q-ToF MS/MS Compound formula is signified by the number of residues of each monosaccharide in the following order: Hex_HexNAc_Fuc_NeuAc_NeuGc. For example, 3_3_0_1_0 is composed of three hexose units, 3 *N*-acetylhexosamine units, and one *N*-acetylneuraminic acid unit.

| Compound | Free | Immobilized |
| --- | --- | --- |
| 3_3_0_0_0* | 799242.78 ± 404059.23 | 21580865.02 ± 6878891.78 |
| 3_3_0_1_0* | 2776214.72 ± 207553.6 | 1332105.27 ± 293538.84 |
| 3_5_0_0_0 | 236666.08 ± 49559.76 | 198854.44 ± 45199.98 |
| 3_5_1_0_0 | 94385.58 ± 19096.3 | 74024.81 ± 10105.03 |
| 3_5_3_0_0* | 86055.4 ± 2201.75 | 30492.32 ± 3491.88 |
| 4_3_0_0_0 | 657800.24 ± 104973.65 | 690113.64 ± 152019.48 |
| 4_3_0_1_0 | 11162362.92 ± 1008355.08 | 7851864.54 ± 1853205.76 |
| 4_3_1_0_0 | 84781.05 ± 50777.72 | 159310.99 ± 19042.66 |
| 4_4_0_0_0 | 48498.23 ± 15617.31 | 52306.48 ± 13897.4 |
| 4_4_0_1_0* | 441455.74 ± 13424.35 | 285918.72 ± 52241.12 |
| 4_4_1_0_0 | 41164.04 ± 4280.51 | 36771.64 ± 10553.2 |
| 4_4_1_1_0 | 147606.91 ± 48540.46 | 85085.56 ± 23598.63 |
| 5_1_0_0_0* | 849481.69 ± 679055.82 | 12453428.49 ± 3091137.69 |
| 5_2_0_1_0 | 472021.56 ± 50533.22 | 408990.12 ± 75505.56 |
| 5_3_0_0_0* | 473556.88 ± 29305.55 | 305769.02 ± 39004.65 |
| 5_3_0_1_0 | 232625.67 ± 46595.19 | 253331.01 ± 21698.76 |
| 5_3_0_0_1* | 137042.62 ± 14987.29 | 71822.15 ± 21631.92 |
| 5_3_1_0_0 | 78083.62 ± 25220.49 | 49889.87 ± 18677.07 |
| 5_3_1_0_1* | 130710.43 ± 19791.8 | 52458.97 ± 14052.17 |
| 5_3_1_1_0 | 29040.36 ± 11127.36 | 25848.68 ± 8785.58 |
| 5_4_0_1_0 | 85183.85 ± 37178.85 | 46257.05 ± 26162.2 |
| 5_4_1_0_0* | 239666.16 ± 16152.18 | 116395.78 ± 13085.38 |
| 6_1_0_0_0* | 1254068.06 ± 986863.26 | 3936276.61 ± 261791.96 |
| 6_2_0_1_0* | 360138.56 ± 10995.21 | 262953.14 ± 35904.76 |
| 6_3_2_0_0* | 129180.71 ± 22445.05 | 52191.15 ± 18783.28 |
| 9_1_0_0_0 | 14047.75 ± 8426.21 | 19782.91 ± 6616.16 |

***** Indicates significant difference between free and immobilized enzyme at *p* < 0.05.
